# Performance Analysis of Centralized Cooperative Schemes for Compressed Sensing

**DOI:** 10.3390/s24020661

**Published:** 2024-01-20

**Authors:** Luca Rugini, Paolo Banelli

**Affiliations:** Department of Engineering, University of Perugia, 06125 Perugia, Italy; paolo.banelli@unipg.it

**Keywords:** compressed sensing, cooperative spectrum sensing, energy detector, number of samples, number of sensors, probability of detection, probability of false alarm, required SNR

## Abstract

This paper presents a performance analysis of centralized spectrum sensing based on compressed measurements. We assume cooperative sensing, where unlicensed users individually perform compressed sensing and send their results to a fusion center, which makes the final decision about the presence or absence of a licensed user signal. Several cooperation schemes are considered, such as and-rule, or-rule, majority voting, soft equal-gain combining (EGC). The proposed analysis provides simplified closed-form expressions that calculate the required number of sensors, the required number of samples, the required compression ratio, and the required signal-to-noise ratio (SNR) as a function of the probability of detection and the probability of the false alarm of the fusion center and of the sensors. The resulting expressions are derived by exploiting some accurate approximations of the test statistics of the fusion center and of the sensors, equipped with energy detectors. The obtained results are useful, especially for a low number of sensors and low sample sizes, where conventional closed-form expressions based on the central limit theorem (CLT) fail to provide accurate approximations. The proposed analysis also allows the self-computation of the performance of each sensor and of the fusion center with reduced complexity.

## 1. Introduction

Cooperative spectrum sensing (CSS) [1,2,3] is a key component of a cognitive radio (CR) network and aims to discover whether some resources are available for signal transmission or not in a given area. Using CSS, unlicensed secondary users (SUs) collaboratively detect the presence or absence of a signal transmitted by a licensed primary user (PU) to understand whether a given channel is already occupied by a PU or free and hence available for SU signal transmission.

One crucial factor of CSS is the amount of data used for sensing purposes. In general, both false alarm (FA) and detection performances improve by increasing the number of SUs and the time spent for sensing at the expense of increased computational processing. Hence, two fundamental parameters are the number of SU sensors and the number of samples used by an SU. Compressed sensing [4,5] is often employed to reduce the number of samples to the reduce complexity. The need for power consumption reduction also requires sensors that are in sleeping mode (i.e., inactive) for a certain fraction of time, to save energy.

A second major factor of CSS is the sensing algorithm of the SUs. The choice of this algorithm mostly depends on the available information about the structure of the PU signal [6,7,8,9,10,11,12,13]. Matched filtering and feature extraction presuppose that the SUs have some knowledge about the PU signal properties. On the other hand, an energy detector (ED) simply monitors the received energy in a specific time–frequency resource, and therefore is more versatile and suitable for those users without knowledge of the PU signal properties. The popularity of the ED is widespread [13,14,15,16,17,18,19,20,21,22,23,24,25,26,27], so that the ED is often also used as a baseline for comparison purposes. Consequently, this paper focuses on SU sensors equipped with EDs.

The third important factor of CSS is the type of cooperation. Indeed, the sensing results acquired by the SUs may be combined either by a centralized fusion center (FC) or using a distributed approach. Herein, we focus on a centralized approach wherein an FC collects the reports of the SUs. In addition, the fusion operation can be performed in several different ways [28,29,30,31,32,33,34,35,36,37,38,39,40,41,42,43,44,45,46,47,48,49,50,51,52]: this paper mainly focuses on *hard reporting (HR)*, where the SUs report their hard decisions to the FC. Anyway, we also consider a *soft reporting (SR)* case, where the SUs report their soft decisions to the FC, which aggregates these values using equal-gain combining (EGC).

Although there exist several performance analyses of CSS with EDs [15,16,18,19,23,24,25,26,27], most of the existing approaches either involve complicated multidimensional functions or have low accuracy. For instance, the exact calculation of the receiver operating characteristic (ROC) of the ED requires the inversion of a chi-squared distribution function [16,19,23,24,25], but this computation can be energy-demanding for low-cost sensors. On the other hand, a popular alternative approach is the Gaussian approximation (GA) for the test statistic of the ED [15,18,26,27]; however, the accuracy of the obtained ROC is reduced, especially when the number of samples is low. Similarly, when the FC applies a *majority-voting (MV) rule*, an exact analysis of the required number of SUs involves complicated functions like the incomplete beta function [53] and its inverse, whereas GA approaches based on the central limit theorem (CLT) may be inaccurate, especially when the number of sensors is low.

This paper aims at closing the gap between the accurate-but-complicated performance analyses and simple-but-inaccurate performance analyses of CSS with EDs. Specifically, this paper proposes simple low-complexity accurate approximations in such a way that the performance assessment can be also self-performed by low-cost sensors and by a low-energy FC. The proposed approximations are obtained by modifying and adapting the methods of probability distribution approximation derived several decades ago [54,55,56,57,58,59,60,61,62,63]. The main contributions of this paper can be summarized as follows.

- For the HR case, we propose closed-form expressions for the number of active sensors required by an FC to achieve a target performance, assuming SUs with a given performance. Several fusion rules are considered: *and-rule*, *or-rule*, and *MV-rule*. In contrast to existing work, the proposed expressions have both accuracy and low complexity.
- For the HR case, we propose closed-form expressions for the number of compressed samples required by an ED to achieve a target performance, assuming a given PU signal-to-noise ratio (SNR). Again, the proposed expressions have both accuracy and low complexity, thereby enabling low-energy self-computation at the SU side. Previous work in [64,65,66] only includes a limited subset of expressions, mainly for conventional non-cooperative sensing, whereas this paper derives a complete performance analysis valid for cooperative compressed sensing.
- For the SR case, we propose closed-form expressions for the aggregate number of samples required by an FC to achieve a target performance. Also, in this case, the proposed expressions combine accuracy with low complexity.

The remainder of this paper is structured as follows. Section 2 outlines the state of the art, while Section 3 introduces the system model. Section 4 develops the approximated performance analysis. Numerical results are presented in Section 5 to validate the proposed approximations, and some concluding remarks are drawn in Section 6.

## 2. State of the Art

In this section, we focus on the literature about CSS performance analysis in terms of the required number of users and required number of samples. For what concerns the number of sensors to warranty (at the FC) a predefined probability of detection and a predefined probability of FA, let us assume HR with *MV-rule* and sensors with the same statistics. In this case, the probabilities of detection and of FA are expressed as a sum [6,15] whose result is expressed by the incomplete beta function [53]. The most common approach for inverting this function is to apply general-purpose software routines, such as in [29,30,33,34,41,45,67,68,69], without an explicit closed-form solution and without complexity analysis. Therefore, the complexity of the FC performance self-estimation may potentially be large, especially when accurate self-estimation is required. A low-complexity approximation applies the CLT, but several other approaches are possible, with greater accuracy and tolerable complexity. For the *MV-rule*, Section 4.1 of this paper will present six low-complexity approximations, whose accuracy will be compared in Section 5.

For what concerns the number of samples required to obtain (at the SU) the desired probabilities of detection and FA, a summary of relevant results is given in [15], which considers two models for the PU signal received by the SUs, namely the deterministic and random models. In the literature, the deterministic model is more popular [17,20,21], and sometimes includes random fading [19,23,25,26] and diversity [9,24]. On the other side, the random model for the PU signal [6] is less common. However, the deterministic model is only appropriate when the SUs are perfectly synchronous and do not apply compressed sensing. When the SU sensors are partially asynchronous or apply compressed sensing, the EDs of the different SUs collect samples that are different in time; therefore, a random PU signal model seems more reasonable. In any case, the results in [15] show that the exact value for the required sample size is significantly different from the CLT-approximated sample size obtained using a Gaussian approach for both deterministic and random models. For the deterministic model, some improved approximations are proposed in [17,20,21], but for the random model, there exist less results [64,65,66]. Section 4.2 of this paper will present three low-complexity approaches, whose accuracy will be compared in Section 5.

## 3. System Model

First, in Section 3.1, we briefly describe the CSS system model without using mathematical equations, in order to give a quick general overview. Second, in Section 3.2, we present a detailed statistical signal model based on mathematical equations, which are instrumental for the performance derivation of the considered CSS scheme in Section 4.

### 3.1. Overview of Centralized Cooperative Sensing

We consider a CR network with multiple sensors acting as SUs and a centralized FC. We assume that the SUs and the FC are located in a specific area. The aim is to sense the presence or absence of a PU signal in a given frequency band. It is assumed that the PU is located outside the area where the SUs and the FC are located. The centralized CSS is performed in three phases. In the first phase, the SUs perform compressed spectrum sensing using an ED. The sensing results of the SUs may be either binary hard decisions or soft decisions. In the second phase, the SUs, or a fraction of them, report their sensing results to the FC, which collects the received results. In the case of sensing results with binary hard decisions, we refer to this second phase as HR. In the case of sensing results with soft decisions, we refer to this second phase as SR. In the third phase, the FC makes a final decision based on the reports received from the SUs. In the case of HR, the FC collects the received binary decisions of the SUs and performs a final decision using a given rule, such as the *and-rule*, the *or-rule*, or the *MV-rule*. In the case of SR, the FC collects the received nonbinary results and performs a soft EGC to obtain the test statistics for the final decision.

### 3.2. Statistical Signal Model

We consider a CR network with *K* SUs equipped with EDs. As in [14], we assume that the PU transmitter is located far away from the area where the *K* SUs are located. We assume that all the SUs have the same activity rate *a*, with 0≤a≤1; hence, 1−a represents the fraction of time of a generic SU staying in sleeping mode. Therefore, the number of active sensors *S* can be expressed as

(1) S=aK.

We assume that the activity rate *a* is chosen in such a way that *S* is always integer. The *S* active sensors perform compressed sensing by observing the received signal in a given bandwidth, while the remaining K−S sensors stay in sleeping mode. The signal vector $x_{i}={\left[x_{1,i},\ldots ,x_{N,i}\right]}^{T}$ received by the *i*th active sensor can be expressed by

(2) $$x_{i}=\alpha s_{i}+w_{i},\;i=1,\ldots ,S,$$

where $s_{i}={\left[s_{1,i},\ldots ,s_{N,i}\right]}^{T}$ is the signal vector sent by the PU received by the *i*th sensor and $w_{i}={\left[w_{1,i},\ldots ,w_{N,i}\right]}^{T}$ represents the noise gathered by the *i*th sensor. The vector dimension *N* in (2) is the sample size, assumed fixed for all the SUs. In (2), α represents the hypothesis on the PU signal with α=0 when the PU signal is absent (hypothesis $H_{0}$) and α=1 when the PU signal is present (Hypothesis $H_{1}$). Both signal and noise are assumed to be circularly symmetric complex Gaussian and white, with zero mean and with covariance $E\left\{s_{i}s_{i}^{H}\right\}=\sigma_{s}^{2}I_{N}$ and $E\left\{w_{i}w_{i}^{H}\right\}=\sigma_{w}^{2}I_{N}$. Since the distance from the PU is similar for all the sensors, $\sigma_{s}^{2}$ is assumed to be equal for all the sensors. In addition, the sensors are of the same type and located in the same area; hence, $\sigma_{w}^{2}$ is assumed to be equal for all the SUs. Originating from different devices, the signal and noise perceived by the *i*th sensor are assumed to be uncorrelated, i.e., $E\left\{s_{i}n_{i}^{H}\right\}=0_{N\times N}$. We also assume that the signal and noise received by different sensors are uncorrelated, i.e., $E\left\{s_{i}n_{l}^{H}\right\}=0_{N\times N}$, $E\left\{s_{i}s_{l}^{H}\right\}=0_{N\times N}$, $E\left\{n_{i}n_{l}^{H}\right\}=0_{N\times N}$, for i≠l. These assumptions are reasonable, because different noise terms are generated by different devices, and the signal terms received by the different SUs are different copies of the same PU signal that is passed through different channels, each one with a different delay and a different phase shift.

The *i*th sensor performs compressed sensing using an M×N compression matrix $\Phi_{i}$, where M<N, as expressed by

(3) $$y_{i}=\Phi_{i}x_{i}=\alpha \Phi_{i}s_{i}+\Phi_{i}w_{i},$$

where $y_{i}={\left[y_{1,i},\ldots ,y_{M,i}\right]}^{T}$ is the compressed vector of the *i*th active sensor. We define the compression ratio c=M/N, with 0<c<1, such that its inverse 1/c represents the undersampling factor from the full sample size *N* to the compressed sample size M=cN. We assume that the compression matrices have $\mathrm{rank}(\Phi_{i})=M$ and satisfy $\Phi_{i}\Phi_{i}^{H}=c^{-1}I_{M}$. A class of matrices that satisfy this condition is the set of the multi-coset samplers [5]. In this case, $\Phi_{i}\Phi_{l}^{H}=0_{M\times M}$ when the multi-coset time delay of the user *i* is different from the user *l*.

The ED of the *i*th active sensor calculates the received signal energy $E_{i}$, expressed by

(4) $$E_{i}=\left|\right|y_{i}{\left|\right|}^{2}=y_{i}^{H}y_{i}=\sum_{m=1}^{M}|y_{m,i}{|}^{2}.$$

In the case of HR, the *i*th active sensor compares $E_{i}$ with a threshold η and produces the binary decision $D_{i}$ expressed by

(5) $$\begin{array}{cc} D_{i} & =1\;\mathrm{if}\;E_{i}\geq \eta , \end{array}$$

(6) $$\begin{array}{cc} D_{i} & =0\;\mathrm{otherwise}. \end{array}$$

Equation (5) is valid when the *i*th ED decides that a PU signal is present, whereas (6) is valid when the *i*th ED decides that a PU signal is absent. We assume that the active sensors use the same threshold η. The choice of this threshold will be discussed in Section 4.2, where useful expressions are derived for the threshold self-computation. After the hard decisions, the *S* active sensors report their binary decisions to the FC. Then, the FC collects the *S* binary decisions and counts them according to

(7) $$T=\sum_{i=1}^{S}D_{i}.$$

Therefore, *T* in (7) represents the number of EDs that detected the presence of a PU signal. The FC makes the final decision on the presence of a PU signal by comparing *T* with a threshold τ, as expressed by

(8) $$\begin{array}{cc} \hat{\alpha }=1 & \;\mathrm{if}\;T\geq \tau , \end{array}$$

(9) $$\begin{array}{cc} \hat{\alpha }=0 & \;\mathrm{otherwise}. \end{array}$$

Equation (8) is valid when the FC decides that a PU signal is present, whereas (9) is valid when the FC decides that a PU signal is absent. Depending on the choice of the FC threshold τ, in Section 4.1, we will consider three cases:

- *And-rule*, where τ=S;
- *Or-rule*, where τ=1;
- *MV-rule*, where τ=(S+1)/2, with *S* being an odd integer.

In the case of SR, we consider the unquantized case where the *i*th active sensor directly sends its computed energy $E_{i}$ to the FC. Then, the FC collects all the energy results using equal-gain combining (EGC), leading to

(10) $$\tilde{T}=\sum_{i=1}^{S}E_{i}=\sum_{i=1}^{S}\sum_{m=1}^{M}|y_{m,i}{|}^{2}.$$

Successively, the FC compares $\tilde{T}$ with a threshold $\tilde{\tau }$ to detect the presence or absence of a PU signal, as in (8) and (9). The choice of $\tilde{\tau }$ will be discussed in Section 4.3. Note that, in this case, the FC acts like a single ED that collects the cumulative energy of the *S* active sensors that act in parallel: this is equivalent to a single ED that uses the SM compressed samples of all the *S* active sensors.

## 4. Performance Analysis

We first consider the HR case by analyzing the FC performance in Section 4.1 and the SU performance in Section 4.2. Successively, we consider the SR case in Section 4.3.

### 4.1. HR Case, FC Performance

In case of HR, the FC receives *S* binary random variables $D_{i}$ from the S active sensors. We assume that these variables are correctly received by the FC. As explained in Section 3.2, the active sensors are EDs with the same threshold and the same noise power; hence, the *S* sensors have the same probability of FA, denoted by $p_{\mathrm{FA}}$. Therefore, each $D_{i}$ is a Bernoulli random variable with $\mathrm{Pr}\left\{D_{i}=1|H_{0}\right\}=p_{\mathrm{FA}}$. The noise samples of different devices are independent, and consequently, under the hypothesis $H_{0}$, the random variable *T* in (7) has a binomial distribution given by

(11) $$\mathrm{Pr}\left\{T=i|H_{0}\right\}=\left(\begin{array}{c} S\\ i \end{array}\right)p_{\mathrm{FA}}^{i}{\left(1-p_{\mathrm{FA}}\right)}^{S-i}.$$

The probability of FA at the FC, denoted by $\pi_{\mathrm{FA}}$, can be calculated as

(12) $$\pi_{\mathrm{FA}}=\mathrm{Pr}\left\{\hat{\alpha }=1|H_{0}\right\}=\sum_{i=\tau }^{S}\left(\begin{array}{c} S\\ i \end{array}\right)p_{\mathrm{FA}}^{i}{\left(1-p_{\mathrm{FA}}\right)}^{S-i}.$$

We now consider the hypothesis $H_{1}$. The *S* sensors are located in the same area, and therefore, the SNR γ, defined as $\gamma =\sigma_{s}^{2}/\sigma_{w}^{2}$, is the same for all the *S* sensors, which have the same probability of detection, denoted by $p_{D}$. This means that each $D_{i}$ is a Bernoulli random variable with $\mathrm{Pr}\left\{D_{i}=1|H_{1}\right\}=p_{D}$. Since the *S* sensors receive the PU signal from different channels, the PU signal samples received from different SUs are assumed to be uncorrelated, i.e., $E\left\{s_{i}s_{l}^{H}\right\}=0_{N\times N}$ for i≠l. Having modeled the signal as Gaussian, the vectors $s_{i}$ and $s_{l}$ are also independent. Consequently, the random variable *T* under the hypothesis $H_{1}$ in (7) also has a binomial distribution given by

(13) $$\mathrm{Pr}\left\{T=i|H_{1}\right\}=\left(\begin{array}{c} S\\ i \end{array}\right)p_{D}^{i}{\left(1-p_{D}\right)}^{S-i}.$$

The probability of detection at the FC, denoted by $\pi_{D}$, can be calculated as

(14) $$\pi_{D}=\mathrm{Pr}\left\{\hat{\alpha }=1|H_{1}\right\}=\sum_{i=\tau }^{S}\left(\begin{array}{c} S\\ i \end{array}\right)p_{D}^{i}{\left(1-p_{D}\right)}^{S-i}.$$

Our aim is to invert (12) and (14) to find the required number of active sensors *S* that warranties a desired performance $\pi_{\mathrm{FA}}$ and $\pi_{D}$ at the FC, for a fixed performance $p_{\mathrm{FA}}$ and $p_{D}$ of the SU sensors.

#### 4.1.1. And-Rule

In the case of the *and-rule*, the FC threshold is τ=S, and hence (12) and (14) become, respectively,

(15) $$\begin{array}{cc} \pi_{\mathrm{FA}} & =\mathrm{Pr}\left\{\hat{\alpha }=1|H_{0}\right\}=\mathrm{Pr}\left\{T=S|H_{0}\right\}=p_{\mathrm{FA}}^{S}, \end{array}$$

(16) $$\begin{array}{cc} \pi_{D} & =\mathrm{Pr}\left\{\hat{\alpha }=1|H_{1}\right\}=\mathrm{Pr}\left\{T=S|H_{1}\right\}=p_{D}^{S}. \end{array}$$

When S>1, both probabilities at the FC are lower than the corresponding probabilities at the SU. Therefore, the *and-rule* is appropriate for an FC that aims to reduce the probability of FA, although this also reduces the probability of detection, with respect to the single SU. Assuming that the FC requires a probability of FA $\pi_{\mathrm{FA}}$ not larger than a target probability ${\bar{\pi }}_{\mathrm{FA}}$, and a probability of detection $\pi_{D}$ not smaller than a target probability ${\bar{\pi }}_{D}$, we obtain that the number of active sensors *S* must satisfy

(17) $$\frac{\ln{\bar{\pi }}_{\mathrm{FA}}}{\ln p_{\mathrm{FA}}}=S_{\min}\leq S\leq S_{\max}=\frac{\ln{\bar{\pi }}_{D}}{\ln p_{D}},$$

where $S_{\min}$ and $S_{\max}$ denote the minimum and the maximum number of active sensors to satisfy the FC performance. Note that (17) implies that $p_{D}\geq {\bar{\pi }}_{D}$. If the probabilities in (17) are chosen incorrectly, it may be impossible to satisfy both inequalities in (17).

Bearing in mind (1), we can use (17) to find the requirement on the number of total sensors *K* (both active and sleeping) for a fixed activity rate *a*, as expressed by

(18) $$\frac{S_{\min}}{a}=K_{\min}\leq K\leq K_{\max}=\frac{S_{\max}}{a},$$

where $S_{\min}$ and $S_{\max}$ are defined in (17). When low-cost sensors want to save energy by staying in sleeping mode more often, their activity rate *a* reduces and consequently a larger number of total sensors *K* is required due to the increase in both limits in (18). Alternatively, if the number of total sensors *K* is fixed, we can find the required activity rate as

(19) $$\frac{S_{\min}}{K}=a_{\min}\leq a\leq a_{\max}=\frac{S_{\max}}{K}.$$

Therefore, if the number of total sensors *K* reduces, the activity rate *a* should increase to warranty the FC performance; in other words, each sensor must stay awake for a longer time.

If we want both *S* and *K* to be fixed (and hence, also the activity rate *a* fixed), to warranty the FC target performance, from (15) and (16), the probability performance of each SU must satisfy

(20) $$\begin{array}{cc} p_{\mathrm{FA}} & \leq \sqrt[{aK}]{{\bar{\pi }}_{\mathrm{FA}}}, \end{array}$$

(21) $$\begin{array}{cc} p_{D} & \geq \sqrt[{aK}]{{\bar{\pi }}_{D}}. \end{array}$$

#### 4.1.2. Or-Rule

In the case of the *or-rule*, the FC threshold is τ=1, and hence (12) and (14) become, respectively

(22) $$\begin{array}{cc} \pi_{\mathrm{FA}} & =\mathrm{Pr}\left\{\hat{\alpha }=1|H_{0}\right\}=1-\mathrm{Pr}\left\{T=0|H_{0}\right\}=1-{\left(1-p_{\mathrm{FA}}\right)}^{S}, \end{array}$$

(23) $$\begin{array}{cc} \pi_{D} & =\mathrm{Pr}\left\{\hat{\alpha }=1|H_{1}\right\}=1-\mathrm{Pr}\left\{T=0|H_{1}\right\}=1-{\left(1-p_{D}\right)}^{S}. \end{array}$$

When S>1, both probabilities at the FC are greater than the corresponding probabilities at the SU. Therefore, the *or-rule* is appropriate for an FC that aims to increase the probability of detection, although this also increases the probability of FA, with respect to the single SU. Assuming that the FC requires a probability of FA $\pi_{\mathrm{FA}}$ that is no larger than a target probability ${\bar{\pi }}_{\mathrm{FA}}$, and a probability of detection $\pi_{D}$ not smaller than a target probability ${\bar{\pi }}_{D}$, we obtain that the number of active sensors *S* must satisfy

(24) $$\frac{\ln(1-{\bar{\pi }}_{D})}{\ln(1-p_{D})}=S_{\min}\leq S\leq S_{\max}=\frac{\ln(1-{\bar{\pi }}_{\mathrm{FA}})}{\ln(1-p_{\mathrm{FA}})}.$$

The main difference of (24) with (17) is the exchanged role of the probabilities of FA, which now determine the maximum number of active sensors $S_{\max}$, and the probabilities of detection, which now determine the minimum number of active sensors $S_{\min}$. This exchanged role can be clarified by defining the corresponding probabilities of missed detection (MD), $p_{\mathrm{MD}}=1-p_{D}$ and $\pi_{\mathrm{MD}}=1-\pi_{D}$, which in this *or-rule* case, play a similar role played by the probabilities of FA when using the *and-rule*.

Again, we can use (24) to find the required number of total sensors *K* for a fixed activity rate *a*, and the required activity rate when *K* is fixed: in both cases, the obtained equations are identical to the corresponding Equations (18) and (19), but with the new definitions of $S_{\min}$ and $S_{\max}$ provided by (24). Also, in this *or-rule* case, when low-cost sensors save energy by frequently staying in sleeping mode, their activity rate *a* is low and consequently a larger number of total sensors *K* is required; vice versa, if the number of total sensors *K* reduces, the activity rate *a* should increase, leading to more power consumption of each SU sensor.

When both *S* and *K* are fixed, to warranty the FC target performance, from (22) and (23), the probability performance of each SU must satisfy

(25) $$\begin{array}{cc} p_{\mathrm{FA}} & \leq 1-\sqrt[{aK}]{1-{\bar{\pi }}_{\mathrm{FA}}}, \end{array}$$

(26) $$\begin{array}{cc} p_{D} & \geq 1-\sqrt[{aK}]{1-{\bar{\pi }}_{D}}. \end{array}$$

#### 4.1.3. MV-Rule

The *MV-rule* uses a threshold τ=(S+1)/2 with *S* odd, and this complicates the inversion of the cumulative binomial functions (12) and (14). To perform the inversion, we make use of the suitable approximations of the binomial distribution, most of which have existed for decades. According to the CLT, a first approach is to use a GA obtained by matching the first two moments, which lead to [54]

(27) $$\begin{array}{cc} \sum_{i=\frac{S+1}{2}}^{S}\left(\begin{array}{c} S\\ i \end{array}\right)p^{i}{\left(1-p\right)}^{S-i} & \approx Q\left(\sqrt{\frac{{\left(1-2p\right)}^{2}}{4p(1-p)}S}\right),\;p<\frac{1}{2}, \end{array}$$

(28) $$\begin{array}{cc} \sum_{i=\frac{S+1}{2}}^{S}\left(\begin{array}{c} S\\ i \end{array}\right)p^{i}{\left(1-p\right)}^{S-i} & \approx 1-Q\left(\sqrt{\frac{{\left(2p-1\right)}^{2}}{4p(1-p)}S}\right),\;p\geq \frac{1}{2}, \end{array}$$

where

(29) $$Q\left(z\right)=\frac{1}{\sqrt{2\pi }}\int_{z}^{+\infty }e^{-t^{2}/2}dt$$

is the right tail of the standard Gaussian probability density function, also known as Gaussian Q-function. Assuming that the FC requires a probability of FA $\pi_{\mathrm{FA}}$ not larger than a target probability ${\bar{\pi }}_{\mathrm{FA}}$, and a probability of detection $\pi_{D}$ that is no smaller than a target probability ${\bar{\pi }}_{D}$ from (12), (14) and (27), we obtain that the number of active sensors *S* must satisfy

(30) $$\begin{array}{cc} S & \geq S_{\min,\mathrm{FA}}=\frac{4p_{\mathrm{FA}}\left(1-p_{\mathrm{FA}}\right)}{{\left(1-2p_{\mathrm{FA}}\right)}^{2}}{\left[Q^{-1}\left({\bar{\pi }}_{\mathrm{FA}}\right)\right]}^{2}, \end{array}$$

(31) $$\begin{array}{cc} S & \geq S_{\min,D}=\frac{4p_{D}\left(1-p_{D}\right)}{{\left(2p_{D}-1\right)}^{2}}{\left[Q^{-1}\left(1-{\bar{\pi }}_{D}\right)\right]}^{2}. \end{array}$$

Therefore, differently from the *and-rule* and the *or-rule*, there is no limit on the maximum number of active sensors. Note that the GA produces the simple results (30) and (31) whose calculation only requires the inverse of the Q-function. It is also worth noting that, when $p_{\mathrm{FA}}=1-p_{D}$ and ${\bar{\pi }}_{\mathrm{FA}}=1-{\bar{\pi }}_{D}$, we have $S_{\min,\mathrm{FA}}=S_{\min,D}$. Bounds on the number of total sensors *K* and on the activity rate *a* can be found by incorporating (1) into (30) and (31), similarly to the derivation of (18) and (19) from (17). Equations (30) and (31) can be used to obtain the maximum probability of FA at the SU and the minimum probability of detection at the SU, for a given number of active SUs *S*, as expressed by

(32) $$\begin{array}{cc} p_{\mathrm{FA}} & \leq \frac{1}{2}\left\{1-\frac{Q^{-1}\left({\bar{\pi }}_{\mathrm{FA}}\right)}{\sqrt{S+{\left[Q^{-1}\left({\bar{\pi }}_{\mathrm{FA}}\right)\right]}^{2}}}\right\}, \end{array}$$

(33) $$\begin{array}{cc} p_{D} & \geq \frac{1}{2}\left\{1+\frac{Q^{-1}\left(1-{\bar{\pi }}_{D}\right)}{\sqrt{S+{\left[Q^{-1}\left(1-{\bar{\pi }}_{D}\right)\right]}^{2}}}\right\}. \end{array}$$

A second possibility for the *MV-rule* is to use an arcsine approximation. According to [55], the most accurate arcsine approximation of a binomial distribution is the one that includes a Borges refinement, as expressed by

(34) $$\begin{array}{cc} \sum_{i=\frac{S+1}{2}}^{S}\left(\begin{array}{c} S\\ i \end{array}\right)p^{i}{\left(1-p\right)}^{S-i} & \approx Q\left(\left[\frac{\pi }{2}-2\arcsin\left(\sqrt{p}\right)\right]\sqrt{S+\frac{1}{3}}\right),\;p<\frac{1}{2}, \end{array}$$

(35) $$\begin{array}{cc} \sum_{i=\frac{S+1}{2}}^{S}\left(\begin{array}{c} S\\ i \end{array}\right)p^{i}{\left(1-p\right)}^{S-i} & \approx 1-Q\left(\left[\frac{\pi }{2}-2\arcsin\left(\sqrt{1-p}\right)\right]\sqrt{S+\frac{1}{3}}\right),\;p\geq \frac{1}{2}, \end{array}$$

which, by (12) and (14), leads to

(36) $$\begin{array}{cc} S & \geq S_{\min,\mathrm{FA}}={\left[\frac{2Q^{-1}\left({\bar{\pi }}_{\mathrm{FA}}\right)}{\pi -4\arcsin(\sqrt{p_{\mathrm{FA}}})}\right]}^{2}-\frac{1}{3}, \end{array}$$

(37) $$\begin{array}{cc} S & \geq S_{\min,D}={\left[\frac{2Q^{-1}\left(1-{\bar{\pi }}_{D}\right)}{\pi -4\arcsin(\sqrt{1-p_{D}})}\right]}^{2}-\frac{1}{3}, \end{array}$$

where we have assumed that the probabilities of FA are lower than one half and the probabilities of detection are greater than one half. As for the GA, Equations (36) and (37) permit the calculations of the maximum probability of FA at the SU and the minimum probability of detection at the SU, for a given number of active SUs *S*, as expressed by

(38) $$\begin{array}{cc} p_{\mathrm{FA}} & \leq \frac{1}{2}\left\{1-\sin\left[\frac{Q^{-1}\left({\bar{\pi }}_{\mathrm{FA}}\right)}{\sqrt{S+\frac{1}{3}}}\right]\right\}, \end{array}$$

(39) $$\begin{array}{cc} p_{D} & \geq \frac{1}{2}\left\{1+\sin\left[\frac{Q^{-1}\left(1-{\bar{\pi }}_{D}\right)}{\sqrt{S+\frac{1}{3}}}\right]\right\}. \end{array}$$

A third invertible approximation of the cumulative binomial function was derived by Camp and Paulson [55], as expressed by

(40) $$\begin{array}{cc} \sum_{i=\frac{S+1}{2}}^{S}\left(\begin{array}{c} S\\ i \end{array}\right)p^{i}{\left(1-p\right)}^{S-i} & \approx Q\left(R\left(p\right)\;\frac{9-\frac{2}{S+1}}{3\sqrt{\frac{2}{S+1}}}\right),\;p<\frac{1}{2}, \end{array}$$

(41) $$\begin{array}{cc} \sum_{i=\frac{S+1}{2}}^{S}\left(\begin{array}{c} S\\ i \end{array}\right)p^{i}{\left(1-p\right)}^{S-i} & \approx 1-Q\left(R\left(1-p\right)\;\frac{9-\frac{2}{S+1}}{3\sqrt{\frac{2}{S+1}}}\right),\;p\geq \frac{1}{2}, \end{array}$$

where R(p) is the ratio defined as

(42) $$R\left(p\right)=\frac{1-{\left(\frac{p}{1-p}\right)}^{1/3}}{\sqrt{1+{\left(\frac{p}{1-p}\right)}^{2/3}}}.$$

We now use (12) with (14) and $p_{\mathrm{FA}}<1/2$ and $p_{D}>1/2$, jointly with (40) and (41). Assuming a probability of FA $\pi_{\mathrm{FA}}<1/2$ at the FC that is no greater than the target probability ${\bar{\pi }}_{\mathrm{FA}}$ and a probability of detection $\pi_{D}>1/2$ at the FC that is no lower than the target probability ${\bar{\pi }}_{D}$, we obtain

(43) $$\begin{array}{cc} \frac{S+\frac{7}{9}}{\sqrt{S+1}} & \geq \frac{\sqrt{2}Q^{-1}\left({\bar{\pi }}_{\mathrm{FA}}\right)}{3R(p_{\mathrm{FA}})}, \end{array}$$

(44) $$\begin{array}{cc} \frac{S+\frac{7}{9}}{\sqrt{S+1}} & \geq \frac{\sqrt{2}Q^{-1}\left(1-{\bar{\pi }}_{D}\right)}{3R(1-p_{D})}, \end{array}$$

which produces the results

(45) $$\begin{array}{cc} S & \geq S_{\min,\mathrm{FA}}=U^{2}\left(p_{\mathrm{FA}},{\bar{\pi }}_{\mathrm{FA}}\right)+U\left(p_{\mathrm{FA}},{\bar{\pi }}_{\mathrm{FA}}\right)\sqrt{U^{2}\left(p_{\mathrm{FA}},{\bar{\pi }}_{\mathrm{FA}}\right)+\frac{4}{9}}-\frac{7}{9}, \end{array}$$

(46) $$\begin{array}{cc} S & \geq S_{\min,D}=U^{2}\left(1\;-\;p_{D},1\;-\;{\bar{\pi }}_{D}\right)+U\left(1\;-\;p_{D},1\;-\;{\bar{\pi }}_{D}\right)\sqrt{U^{2}\left(1\;-\;p_{D},1\;-\;{\bar{\pi }}_{D}\right)+\frac{4}{9}}-\frac{7}{9}, \end{array}$$

where

(47) $$U\left(p,\bar{\pi }\right)=\frac{Q^{-1}\left(\bar{\pi }\right)}{3R(p)},$$

where $Q^{-1}\left(z\right)$ is the inverse of (29) and R(p) is defined in (42).

The three methods discussed so far (Gaussian, arcsine, and Camp–Paulson approximations) directly approximate the binomial distribution. Another set of approximations can be obtained by exploiting the relation between the binomial distribution and the incomplete beta function, as expressed in Equation 26.5.24 of [53]:

(48) $$\sum_{i=\tau }^{S}\left(\begin{array}{c} S\\ i \end{array}\right)p^{i}{\left(1-p\right)}^{S-i}=I_{p}\left(\tau ,S-\tau +1\right),$$

where $I_{p}\left(z_{1},z_{2}\right)$ is the (normalized) incomplete beta function defined as [53]

(49) $$I_{p}\left(z_{1},z_{2}\right)=\frac{1}{B(z_{1},z_{2})}\int_{0}^{p}t^{z_{1}-1}{\left(1-t\right)}^{z_{2}-1}dt,$$

where $B(z_{1},z_{2})$ is the beta function [53]. The *MV-rule* uses a threshold τ=(S+1)/2 with *S* odd: this leads to $I_{p}\left(\frac{S+1}{2},\frac{S+1}{2}\right)$ in (48), for which several invertible approximations exist [53,56,57,58,59]. The method of Fisher [56,57] uses the approximation

(50) $$\begin{array}{cc} \sum_{i=\frac{S+1}{2}}^{S}\left(\begin{array}{c} S\\ i \end{array}\right)p^{i}{\left(1-p\right)}^{S-i} & \approx Q\left(L\left(p\right)\sqrt{S}\right),\;p<\frac{1}{2}, \end{array}$$

(51) $$\begin{array}{cc} \sum_{i=\frac{S+1}{2}}^{S}\left(\begin{array}{c} S\\ i \end{array}\right)p^{i}{\left(1-p\right)}^{S-i} & \approx 1-Q\left(L\left(1-p\right)\sqrt{S}\right),\;p\geq \frac{1}{2}, \end{array}$$

where L(p) is defined as

(52) $$L\left(p\right)=\frac{1}{2}\ln\frac{1-p}{p}.$$

Using (12) and (14) with (50) and (51), and assuming $p_{\mathrm{FA}}<1/2$, $p_{D}>1/2$, $\pi_{\mathrm{FA}}\leq {\bar{\pi }}_{\mathrm{FA}}<1/2$ and $\pi_{D}\geq {\bar{\pi }}_{D}>1/2$, we obtain

(53) $$\begin{array}{cc} S & \geq S_{\min,\mathrm{FA}}={\left[\frac{Q^{-1}\left({\bar{\pi }}_{\mathrm{FA}}\right)}{L(p_{\mathrm{FA}})}\right]}^{2}, \end{array}$$

(54) $$\begin{array}{cc} S & \geq S_{\min,D}={\left[\frac{Q^{-1}\left(1-{\bar{\pi }}_{D}\right)}{L(1-p_{D})}\right]}^{2}. \end{array}$$

If we assume a given number of active sensors *S*, the maximum probability of FA required at the SU and the minimum probability of detection required at the SU are given by

(55) $$\begin{array}{cc} p_{\mathrm{FA}} & \leq \frac{1}{1+\exp\{2Q^{-1}\left({\bar{\pi }}_{\mathrm{FA}}\right)/\sqrt{S}\}}, \end{array}$$

(56) $$\begin{array}{cc} p_{D} & \geq \frac{\exp\{2Q^{-1}\left(1-{\bar{\pi }}_{D}\right)/\sqrt{S}\}}{1+\exp\{2Q^{-1}\left(1-{\bar{\pi }}_{D}\right)/\sqrt{S}\}}, \end{array}$$

where ${\bar{\pi }}_{\mathrm{FA}}$ and ${\bar{\pi }}_{D}$ are the FC target probability of FA and the FC target probability of detection, respectively.

On the other hand, the method of Cochran [56,57] refines the method of Fisher, such that the approximation of the binomial sum becomes

(57) $$\begin{array}{cc} \sum_{i=\frac{S+1}{2}}^{S}\left(\begin{array}{c} S\\ i \end{array}\right)p^{i}{\left(1-p\right)}^{S-i} & \approx Q\left(\frac{L(p)}{\sqrt{1+\frac{1}{6}L^{2}\left(p\right)}}\sqrt{S+\frac{1}{2}}\right),\;p<\frac{1}{2}, \end{array}$$

(58) $$\begin{array}{cc} \sum_{i=\frac{S+1}{2}}^{S}\left(\begin{array}{c} S\\ i \end{array}\right)p^{i}{\left(1-p\right)}^{S-i} & \approx 1-Q\left(\frac{L(1-p)}{\sqrt{1+\frac{1}{6}L^{2}\left(1-p\right)}}\sqrt{S+\frac{1}{2}}\right),\;p\geq \frac{1}{2}, \end{array}$$

where L(p) is defined in (52). Note that, from (52), L(1−p)=−L(p), hence $L^{2}\left(1-p\right)=L^{2}\left(p\right)$. Using the same assumptions $p_{\mathrm{FA}}<1/2$, $p_{D}>1/2$, $\pi_{\mathrm{FA}}\leq {\bar{\pi }}_{\mathrm{FA}}<1/2$ and $\pi_{D}\geq {\bar{\pi }}_{D}>1/2$, (57) and (58) jointly with (12) and (14) lead to the following bounds for the minimum number of active sensors:

(59) $$\begin{array}{cc} S & \geq S_{\min,\mathrm{FA}}={\left[\frac{Q^{-1}\left({\bar{\pi }}_{\mathrm{FA}}\right)}{L(p_{\mathrm{FA}})}\right]}^{2}+\frac{{\left[Q^{-1}\left({\bar{\pi }}_{\mathrm{FA}}\right)\right]}^{2}}{6}-\frac{1}{2}, \end{array}$$

(60) $$\begin{array}{cc} S & \geq S_{\min,D}={\left[\frac{Q^{-1}\left(1-{\bar{\pi }}_{D}\right)}{L(1-p_{D})}\right]}^{2}+\frac{{\left[Q^{-1}\left(1-{\bar{\pi }}_{D}\right)\right]}^{2}}{6}-\frac{1}{2}. \end{array}$$

In this case, the required probability of FA and the required probability of detection at the SU, assuming a fixed number of active sensors *S*, are

(61) $$\begin{array}{cc} p_{\mathrm{FA}} & \leq \frac{1}{1+\exp\left\{2Q^{-1}\left({\bar{\pi }}_{\mathrm{FA}}\right)/\sqrt{S-\frac{1}{6}\left\{{\left[Q^{-1}\left({\bar{\pi }}_{\mathrm{FA}}\right)\right]}^{2}-3\right\}}\right\}}, \end{array}$$

(62) $$\begin{array}{cc} p_{D} & \geq \frac{\exp\left\{2Q^{-1}\left(1\;-\;{\bar{\pi }}_{D}\right)/\sqrt{S-\frac{1}{6}\left\{{\left[Q^{-1}\left(1\;-\;{\bar{\pi }}_{D}\right)\right]}^{2}-3\right\}}\right\}}{1+\exp\left\{2Q^{-1}\left(1\;-\;{\bar{\pi }}_{D}\right)/\sqrt{S-\frac{1}{6}\left\{{\left[Q^{-1}\left(1\;-\;{\bar{\pi }}_{D}\right)\right]}^{2}-3\right\}}\right\}}, \end{array}$$

where ${\bar{\pi }}_{\mathrm{FA}}$ and ${\bar{\pi }}_{D}$ are again the target probabilities (of FA and of detection, respectively) required at the FC.

Another approximation is the one proposed by Carter [58,59] which, with our notation, can be expressed as

(63) $$\begin{array}{cc} \sum_{i=\frac{S+1}{2}}^{S}\left(\begin{array}{c} S\\ i \end{array}\right)p^{i}{\left(1\;-\;p\right)}^{S-i} & \approx Q\left(\sqrt{3\left[\sqrt{{\left(S\;-\;\frac{1}{2}\right)}^{2}+\frac{2}{3}L^{2}\left(p\right)S^{2}}-S+\frac{1}{2}\right]}\right),\;p<\frac{1}{2}, \end{array}$$

(64) $$\begin{array}{cc} \sum_{i=\frac{S+1}{2}}^{S}\left(\begin{array}{c} S\\ i \end{array}\right)p^{i}{\left(1\;-\;p\right)}^{S-i} & \approx 1\;-\;Q\left(\sqrt{3\left[\sqrt{{\left(S\;-\;\frac{1}{2}\right)}^{2}\;+\;\frac{2}{3}L^{2}\left(p\right)S^{2}}-S+\frac{1}{2}\right]}\right),\;p\geq \frac{1}{2}, \end{array}$$

where L(p) is defined in (52). In (64), we used $L^{2}\left(p\right)=L^{2}\left(1-p\right)$. Using again $p_{\mathrm{FA}}<1/2$, $p_{D}>1/2$, $\pi_{\mathrm{FA}}\leq {\bar{\pi }}_{\mathrm{FA}}<1/2$ and $\pi_{D}\geq {\bar{\pi }}_{D}>1/2$, we can join (12) and (14) with (63) and (64) to obtain the minimum number of required sensors expressed by

(65) $$\begin{array}{cc} S & \geq S_{\min,\mathrm{FA}}=\frac{1}{2}{\left[\frac{Q^{-1}\left({\bar{\pi }}_{\mathrm{FA}}\right)}{L(p_{\mathrm{FA}})}\right]}^{2}\left\{1+\sqrt{1+2L^{2}\left(p_{\mathrm{FA}}\right)\left\{\frac{1}{3}-\frac{1}{{\left[Q^{-1}\left({\bar{\pi }}_{\mathrm{FA}}\right)\right]}^{2}}\right\}}\right\}, \end{array}$$

(66) $$\begin{array}{cc} S & \geq S_{\min,D}=\frac{1}{2}{\left[\frac{Q^{-1}\left(1\;-\;{\bar{\pi }}_{D}\right)}{L(1\;-\;p_{D})}\right]}^{2}\left\{1+\sqrt{1+2L^{2}\left(p_{D}\right)\left\{\frac{1}{3}-\frac{1}{{\left[Q^{-1}\left(1\;-\;{\bar{\pi }}_{D}\right)\right]}^{2}}\right\}}\right\}, \end{array}$$

where ${\bar{\pi }}_{\mathrm{FA}}$ and ${\bar{\pi }}_{D}$ are the target probabilities required at the FC. When the number of active sensors *S* is fixed, the required performance of the SU sensors can be expressed by

(67) $$\begin{array}{cc} p_{\mathrm{FA}} & \leq \frac{1}{1+\exp\left\{2Q^{-1}\left({\bar{\pi }}_{\mathrm{FA}}\right)\sqrt{S+\frac{1}{6}\left\{{\left[Q^{-1}\left({\bar{\pi }}_{\mathrm{FA}}\right)\right]}^{2}-3\right\}}/S\right\}}, \end{array}$$

(68) $$\begin{array}{cc} p_{D} & \geq \frac{\exp\left\{2Q^{-1}\left(1\;-\;{\bar{\pi }}_{D}\right)\sqrt{S+\frac{1}{6}\left\{{\left[Q^{-1}\left(1\;-\;{\bar{\pi }}_{D}\right)\right]}^{2}-3\right\}}/S\right\}}{1+\exp\left\{2Q^{-1}\left(1\;-\;{\bar{\pi }}_{D}\right)\sqrt{S+\frac{1}{6}\left\{{\left[Q^{-1}\left(1\;-\;{\bar{\pi }}_{D}\right)\right]}^{2}-3\right\}}/S\right\}}. \end{array}$$

Summarizing, for the *MV-rule*, we presented six approximations, and each one gives a couple of lower bounds on the minimum number of required active sensors *S* for a target performance at the FC, expressed by (30) and (31), (36) and (37), (45) and (46), (53) and (54), (59) and (60), as well as (65) and (66). The two bounds in each couple become equal $\left(S_{\min,\mathrm{FA}}=S_{\min,D}\right)$ when $p_{D}=1-p_{\mathrm{FA}}$ and ${\bar{\pi }}_{D}=1-{\bar{\pi }}_{\mathrm{FA}}$. Similar bounds on the minimum number of required total sensors and on the activity rate can be obtained using (1). The accuracy of the above six approximations is compared in Section 5, using numerical examples.

### 4.2. HR Case, SU Performance

Section 4.1 has detailed the performance of the FC that receive HR information from the SU, and proposed analytical expressions that linked the probability of FA (and of detection) of the FC to the number of sensors and to the probability of FA (and of detection) of the active sensors. In contrast, this Section 4.2 focuses on the performance of the SU sensors with HR and provides analytical expressions that link the probability of FA (and of detection) of the SU sensor to the number of sensors and to the SNR.

Assuming that the *i*th SU is equipped with an ED, under the hypothesis $H_{0}$, the energy $E_{i}$ in (4) is a chi-squared random variable, with a number of degrees of freedom equal to 2M=2cN, where *N* is the total number of samples and *c* is the compression ratio. Therefore, according to (5), the probability of FA of the SU sensor can be expressed as

(69) $$p_{\mathrm{FA}}=\mathrm{Pr}\left\{D_{i}=1|H_{0}\right\}=1-F_{2M}\left(\frac{2\eta }{\sigma_{w}^{2}}\right),$$

where

(70) $$F_{2M}\left(z\right)=\frac{1}{\Gamma (M)}\int_{0}^{z/2}t^{M-1}e^{-t}dt,$$

where Γ(M) denotes the gamma function [53]. According to (69), the ED threshold is expressed by

(71) $$\eta =\frac{\sigma_{w}^{2}}{2}F_{2M}^{-1}\left(1-p_{\mathrm{FA}}\right),$$

where $F_{2M}^{-1}\left(z\right)$ is the inverse function of $F_{2M}\left(z\right)$ with respect to the argument under parentheses.

Under the hypothesis $H_{1}$, the energy $E_{i}$ in (4) is a chi-squared random variable with the same number of degrees of freedom than under $H_{0}$, but with increased power due to the presence of a PU signal. Therefore, according to (5), the probability of detection of the SU sensor can be expressed as

(72) $$p_{D}=\mathrm{Pr}\left\{D_{i}=1|H_{1}\right\}=1-F_{2M}\left(\frac{2\eta }{\sigma_{s}^{2}+\sigma_{w}^{2}}\right).$$

By inserting (71) into (72), we obtain the receiver operating characteristic (ROC) of the SU sensor as

(73) $$p_{D}=1-F_{2M}\left(\frac{F_{2M}^{-1}\left(1-p_{\mathrm{FA}}\right)}{1+\gamma }\right),$$

where $\gamma =\sigma_{s}^{2}/\sigma_{w}^{2}$ is the sensing SNR.

Our aim is to invert (73) to find the required number of samples *N* that warranties a desired performance $p_{\mathrm{FA}}$ and $p_{D}$ at the SU for a fixed SNR. In principle, we could use an iterative algorithm to find the minimum compressed sample size *M* that warranties the SU performance, and then calculate the required sample size as N=M/c. However, this iterative algorithm would require the multiple evaluation of integral functions like $F_{2M}\left(z\right)$ and $F_{2M}^{-1}\left(z\right)$; hence, this approach is not appropriate for low-complexity SU sensors that want to self-calculate the required sample size. Therefore, whenever possible, we look for invertible low-complexity accurate approximations of the ROC (73). When iterative algorithms are unavoidable, we resort to the low-complexity approximations of $F_{2M}\left(z\right)$ and $F_{2M}^{-1}\left(z\right)$. Specifically, we consider the three following approaches.

- *Standard GA*, where a chi-squared random variable is approximated by a Gaussian random variable using the CLT (see [15] and references therein).
- *Power-of-Gaussian approximation (PGA)*, where a chi-squared random variable is approximated by the power of a Gaussian random variable [60,61,64], with a suitable power exponent.
- *Polynomial approximation (PA)*, where a polynomial of the *r*th root of a chi-squared random variable is approximated by a Gaussian random variable [62,63], using suitable degree and coefficients.

A comparison of these three approaches for non-cooperative non-compressed sensing was introduced in the conference paper [65]. Herein, we extend the earlier preliminary results of [65,66] and derive a complete performance analysis valid for all three approaches when applied to compressed sensing.

#### 4.2.1. Standard GA

The standard GA can be obtained by approximating (70) as [15]

(74) $$F_{2M}\left(z\right)\approx 1-Q\left(\left[\frac{z}{2M}-1\right]\sqrt{M}\right),$$

which leads to the approximated inverse function

(75) $$F_{2M}^{-1}\left(z\right)\approx 2M\left[\frac{Q^{-1}\left(1-z\right)}{\sqrt{M}}+1\right].$$

Equations (75) and (71) allow the ED to self-compute the threshold as

(76) $$\eta \approx M\sigma_{w}^{2}\left[\frac{Q^{-1}\left(p_{\mathrm{FA}}\right)}{\sqrt{M}}+1\right].$$

Note that the approximated expression (76) is much simpler than the exact expression (71), because the inverse Q-function is a one-dimensional function that is easier to compute than $F_{2M}^{-1}\left(1-p_{\mathrm{FA}}\right)$. Equation (74) can be combined with (73) to obtain the performance of the SU sensor: when the number of samples *N* and the SNR γ are fixed, (73) and (74) lead to the GA ROC

(77) $$p_{D}\approx Q\left(\frac{Q^{-1}\left(p_{\mathrm{FA}}\right)-\gamma \sqrt{cN}}{1+\gamma }\right).$$

When the SU probabilities of FA $p_{\mathrm{FA}}$ and detection $p_{D}$ are fixed, from (77), we obtain the required number of samples *N* as a function of the SNR γ and of the compression ratio *c*, as expressed by

(78) $$N\geq \frac{1}{c}{\left[\frac{Q^{-1}\left(p_{\mathrm{FA}}\right)-\left(1+\gamma \right)Q^{-1}\left(p_{D}\right)}{\gamma }\right]}^{2}.$$

Moreover, when the SU probabilities of FA $p_{\mathrm{FA}}$ and detection $p_{D}$ are fixed, from (77) we obtain the required SNR γ as a function of the sample size *N* and of the compression ratio *c*, as expressed by

(79) $$\gamma \geq \frac{Q^{-1}\left(p_{\mathrm{FA}}\right)-Q^{-1}\left(p_{D}\right)}{Q^{-1}\left(p_{D}\right)+\sqrt{cN}}.$$

#### 4.2.2. PGA

In general, GA approaches can only be accurate in the middle of the Gaussian shape, or when the number of samples is large. To improve the accuracy near the tails of a probability density function (pdf), PGA approaches can be used, especially when the number of samples is low. In a nutshell, PGA approaches replace a chi-squared random variable with the *r*th power of a Gaussian random variable, with *r* integer. These PGA approaches are also called root transformations, because a Gaussian random variable approximates the *r*th root of a chi-squared random variable. When r=1, PGA reduces to GA. The improved accuracy of PGA approaches with r>1 has been proven in [61] and in [62]. Specifically, [61] has shown that the Kullback–Leibler divergence between the pdf of the *r*th root of a chi-squared random variable and the Gaussian pdf is minimized for 3≤r≤4; in addition, [62] has compared the cumulants of the two pdfs, showing that the best match is obtained when 2≤r≤4. Therefore, it is expected that a performance analysis based on a PGA approach will be more accurate than a performance analysis based on the GA.

The PGA can be obtained by approximating (70) as [60]

(80) $$F_{2M}\left(z\right)\approx 1-Q\left(\left[\sqrt[{r}]{\frac{z}{2M}}-\frac{2Mr^{2}-r+1}{2Mr^{2}}\right]r\sqrt{M}\right),$$

which can be used in place of (74) to improve the accuracy. In this case, the inverse function is approximated as

(81) $$F_{2M}^{-1}\left(z\right)\approx 2M{\left[\frac{Q^{-1}\left(1-z\right)}{r\sqrt{M}}+\frac{2Mr^{2}-r+1}{2Mr^{2}}\right]}^{r}.$$

Therefore, combining (81) with (71), the ED can easily self-compute its threshold as

(82) $$\eta \approx M\sigma_{w}^{2}{\left[\frac{Q^{-1}\left(p_{\mathrm{FA}}\right)}{r\sqrt{M}}+\frac{2Mr^{2}-r+1}{2Mr^{2}}\right]}^{r},$$

which coincides with (76) when r=1. Equation (80) can be combined with (73) to obtain the performance of the SU sensor: when the number of samples *N* and the SNR γ are fixed, after some calculus, (73) and (80) lead to the PGA ROC as

(83) $$p_{D}\approx Q\left(\frac{Q^{-1}\left(p_{\mathrm{FA}}\right)-\left(\sqrt[{r}]{1+\gamma }-1\right)\left(r\sqrt{cN}-\frac{r-1}{2r\sqrt{cN}}\right)}{\sqrt[{r}]{1+\gamma }}\right).$$

When r=1, (83) reduces to (77). When the SU probabilities of FA $p_{\mathrm{FA}}$ and detection $p_{D}$ are fixed, from (83), we obtain the required number of samples *N* as a function of the SNR γ and of the compression ratio *c*, as expressed by

(84) $$N\geq \frac{1}{c}\left(b_{r}+\frac{r-1}{2r^{2}}+\sqrt{b_{r}^{2}+\frac{r-1}{r^{2}}b_{r}}\right),$$

where

(85) $$b_{r}=\frac{1}{2}{\left[\frac{Q^{-1}\left(p_{\mathrm{FA}}\right)-\sqrt[{r}]{1+\gamma }Q^{-1}\left(p_{D}\right)}{r(\sqrt[{r}]{1+\gamma }-1)}\right]}^{2}.$$

In addition, when the SU probabilities of FA $p_{\mathrm{FA}}$ and detection $p_{D}$ are fixed, from (83), we obtain the required SNR γ as a function of the sample size *N* and of the compression ratio *c* as expressed by

(86) $$\gamma \geq {\left[\frac{Q^{-1}\left(p_{\mathrm{FA}}\right)+r\sqrt{cN}-\frac{r-1}{2r\sqrt{cN}}}{Q^{-1}\left(p_{D}\right)+r\sqrt{cN}-\frac{r-1}{2r\sqrt{cN}}}\right]}^{r}-1.$$

When r=1, (84) reduces to (78), while (86) boils down to (79).

#### 4.2.3. PA

PA approaches try to increase the accuracy of PGA [62,63] by constructing a polynomial expression whose variable is the *r*th root of a chi-squared random variable; then, this polynomial is approximated by a Gaussian random variable. Therefore, a PA approach is also called a linear combination of power (or root) transformations.

A first PA, originally developed by Goria [62], is obtained by first defining the fourth root transformation

(87) $$x=\sqrt[{4}]{\frac{z}{2M}},$$

where *z* is a chi-squared random variable with 2M degrees of freedom, and then choosing the polynomial expression

(88) $$g=x^{2}+4x.$$

Successively, *g* in (88) is approximated as a Gaussian random variable with a mean $5-\frac{1}{2M}$ and variance $\frac{9}{4M}$. Consequently, the chi-squared distribution (70) is approximated as

(89) $$F_{2M}\left(z\right)\approx 1-Q\left(\left[\sqrt{\frac{z}{2M}}+4\sqrt[{4}]{\frac{z}{2M}}-\left(5-\frac{1}{2M}\right)\right]\frac{2}{3}\sqrt{M}\right),$$

which can be inverted with respect to *z* as

(90) $$F_{2M}^{-1}\left(z\right)\approx 2Mv^{4}\left(1-z,M\right),$$

where

(91) $$v\left(z,M\right)=\sqrt{\frac{3}{2\sqrt{M}}Q^{-1}\left(z\right)+9-\frac{1}{2M}}-2.$$

Hence, using (90) together with (71), the threshold can be computed by the SU as

(92) $$\eta \approx M\sigma_{w}^{2}v^{4}\left(p_{\mathrm{FA}},M\right).$$

Note that (92) is much easier to compute than (71). From (73), the approximated ROC is given by

(93) $$p_{D}\approx Q\left(\frac{2\sqrt{cN}}{3}\left[\frac{v^{2}\left(p_{\mathrm{FA}},cN\right)}{\sqrt{1+\gamma }}+\frac{4v(p_{\mathrm{FA}},cN)}{\sqrt[{4}]{1+\gamma }}-5+\frac{1}{2cN}\right]\right).$$

When the SU probabilities of FA $p_{\mathrm{FA}}$ and detection $p_{D}$ are fixed, from (93), we obtain the required SNR γ as a function of the sample size *N* and of the compression ratio *c*, as expressed by

(94) $$\gamma \geq {\left[\frac{v(p_{\mathrm{FA}},cN)}{v(p_{D},cN)}\right]}^{4}-1,$$

where $v(p_{\mathrm{FA}},cN)$ and $v(p_{D},cN)$ are calculated using (91).

When $p_{\mathrm{FA}}$, $p_{D}$, and the SNR γ are fixed, the approximated ROC (93) can also be used to obtain the required number of samples *N* as a function of the compression ratio *c*. A closed-form expression for the required sample size *N* would be complicated; therefore, we resort to an iterative algorithm. Since N=M/c, the iterative algorithm looks for the minimum compressed sample size *M* that satisfies a given $p_{\mathrm{FA}}$ and a given $p_{D}$. The algorithm starts with a tentative value M=1, and calculates two possible thresholds $\eta_{\mathrm{FA}}$ and $\eta_{D}$, as expressed by

(95) $$\begin{array}{cc} \eta_{\mathrm{FA}} & =M\sigma_{w}^{2}v^{4}\left(p_{\mathrm{FA}},M\right), \end{array}$$

(96) $$\begin{array}{cc} \eta_{D} & =M\sigma_{w}^{2}\left(1+\gamma \right)v^{4}\left(p_{D},M\right), \end{array}$$

where the function v(z,M) is defined in (91). Equation (95) represents the minimum value of the ED threshold to warranty a probability of FA $p_{\mathrm{FA}}$ using *M* samples, while (96) is the maximum value of the ED threshold to warranty a probability of detection $p_{D}$ using *M* samples. If $\eta_{\mathrm{FA}}\leq \eta_{D}$, then any threshold η in the interval $\eta_{\mathrm{FA}}\leq \eta \leq \eta_{D}$ satisfies both probability requirements; hence, the tentative *M* is a sufficient number for the required compressed sample size. Instead, if $\eta_{\mathrm{FA}}>\eta_{D}$, then it is impossible to find a unique threshold η that satisfies both probability requirements, and hence the tentative *M* is insufficient. Therefore, when $\eta_{\mathrm{FA}}>\eta_{D}$, the iterative algorithm doubles the tentative value of *M*, and performs a new computation of (95) and (96). The two updated thresholds are compared again, and, if $\eta_{\mathrm{FA}}>\eta_{D}$ again, then the algorithm doubles again the tentative value of *M*. After a finite number of doublings, the algorithm finds a value of $M=M_{\max}$ such that $\eta_{\mathrm{FA}}\leq \eta_{D}$, and this value can be used to set an upper bound $N_{\max}=M_{\max}/c$ on the required sample size. At this point, the minimum value of *M* that satisfies both probability requirements surely lies in the interval $M_{\max}/2<M\leq M_{\max}$, so the iterative algorithm can use an interval-halving method to refine the search. Consequently, the algorithm sets a new tentative value $M=3M_{\max}/4$, which is in the midway of the interval $M_{\max}/2<M\leq M_{\max}$, and updates the two thresholds (95) and (96) again. If $\eta_{\mathrm{FA}}>\eta_{D}$, then the new search interval reduces to $3M_{\max}/4<M\leq M_{\max}$, whereas, if $\eta_{\mathrm{FA}}\leq \eta_{D}$, then the new search interval reduces to $M_{\max}/2<M\leq 3M_{\max}/4$. Then, the algorithm continues again by selecting a tentative *M* in the midway of the resulting interval, and by updating the two thresholds. The iterative algorithm ends when the size of the search interval becomes equal to one, say $M_{\mathrm{final}}-1<M\leq M_{\mathrm{final}}$, yielding the compressed sample size $M=M_{\mathrm{final}}$ as the final solution. At the end of the iterative algorithm, the required sample size is set to $N=M_{\mathrm{final}}/c$. It is noteworthy that the iterative algorithm has logarithmic complexity with respect to $M_{\mathrm{final}}$: the number of iterations is upper bounded by $2\lceil \log_{2}\left(M_{\mathrm{final}}\right)\rceil$, where ⌈x⌉ is the integer ceiling function, because the number of doublings is upper bounded by $\left\lceil \log_{2}\left(M_{\mathrm{final}}\right)\right\rceil$, while the number of halvings is upper bounded by $\lceil \log_{2}\left(M_{\mathrm{final}}\right)\rceil -1$.

A second PA, originally developed by Canal [63], is obtained by first defining the sixth-root transformation

(97) $$x=\sqrt[{6}]{\frac{z}{2M}}$$

and then choosing the polynomial

(98) $$g=\frac{x^{3}}{3}-\frac{x^{2}}{2}+x.$$

Successively, *g* in (98) is approximated as a Gaussian random variable with the mean $\frac{5}{6}-\frac{1}{18M}$ and variance $\frac{1}{36M}$. Consequently, the chi-squared distribution (70) is approximated as

(99) $$F_{2M}\left(z\right)\approx 1-Q\left(\left[\frac{1}{3}\sqrt{\frac{z}{2M}}-\frac{1}{2}\sqrt[{3}]{\frac{z}{2M}}+\sqrt[{6}]{\frac{z}{2M}}-\left(\frac{5}{6}-\frac{1}{18M}\right)\right]6\sqrt{M}\right),$$

which can be inverted, using Cardano’s method for cubic equations, as

(100) $$F_{2M}^{-1}\left(z\right)\approx 2Mq^{6}\left(1-z,M\right),$$

where

(101) $$\begin{array}{cc} q(z,M) & =\frac{1\;+\;\sqrt[{3}]{\sqrt{{\bar{q}}^{2}\left(z,M\right)\;+\;27}\;+\;\bar{q}\left(z,M\right)}\;-\;\sqrt[{3}]{\sqrt{{\bar{q}}^{2}\left(z,M\right)\;+\;27}\;-\;\bar{q}\left(z,M\right)}}{2}, \end{array}$$

(102) $$\begin{array}{cc} \bar{q}\left(z,M\right) & =\frac{2}{\sqrt{M}}Q^{-1}\left(z\right)+5-\frac{2}{3M}. \end{array}$$

Consequently, using (71) and (100), in this second case, the ED can obtain the threshold as

(103) $$\eta \approx M\sigma_{w}^{2}q^{6}\left(p_{\mathrm{FA}},M\right).$$

From (73), the approximated ROC is given by

(104) $$p_{D}\approx Q\left(6\sqrt{cN}\left[\frac{q^{3}\left(p_{\mathrm{FA}},cN\right)}{3\sqrt{1+\gamma }}-\frac{q^{2}\left(p_{\mathrm{FA}},cN\right)}{2\sqrt[{3}]{1+\gamma }}+\frac{q(p_{\mathrm{FA}},cN)}{\sqrt[{6}]{1+\gamma }}-\frac{5}{6}+\frac{1}{18cN}\right]\right).$$

When the SU probabilities of FA $p_{\mathrm{FA}}$ and detection $p_{D}$ are fixed, (104) gives the required SNR γ as a function of the sample size *N* and of the compression ratio *c*, as expressed by

(105) $$\gamma \geq {\left[\frac{q(p_{\mathrm{FA}},cN)}{q(p_{D},cN)}\right]}^{6}-1,$$

where $q(p_{\mathrm{FA}},cN)$ and $q(p_{D},cN)$ are calculated using (101) and (102). When $p_{\mathrm{FA}}$, $p_{D}$, and the SNR γ are fixed, the approximated ROC (104) can be used to obtain the required number of samples *N* as a function of the compression ratio *c*, using the same iterative algorithm detailed above, provided that the two thresholds $\eta_{\mathrm{FA}}$ and $\eta_{D}$ are updated using

(106) $$\begin{array}{cc} \eta_{\mathrm{FA}} & =M\sigma_{w}^{2}q^{6}\left(p_{\mathrm{FA}},M\right), \end{array}$$

(107) $$\begin{array}{cc} \eta_{D} & =M\sigma_{w}^{2}\left(1+\gamma \right)q^{6}\left(p_{D},M\right), \end{array}$$

instead of (95) and (96).

### 4.3. SR Case, FC Performance

This section considers the SR case, where the SUs do not make any hard decision and transmit soft information to the FC, specifically the energy values collected by the EDs. According to (10), the FC combines the received energy values using EGC, therefore the FC acts like a single ED with MS samples, where *S* is the number of active SUs and *M* is the compressed sample size of each ED.

Under the hypothesis $H_{0}$, the variable $\tilde{T}$ in (10) is a chi-squared random variable with 2MS=2acKN degrees of freedom, where *a* is the activity factor, *c* is the compression ratio, *K* is the total number of users, and *N* is the total number of samples of each ED. Therefore, the probability of FA of the FC can be expressed as

(108) $$\pi_{\mathrm{FA}}=\mathrm{Pr}\left\{\tilde{T}\geq \tilde{\tau }|H_{0}\right\}=1-F_{2acKN}\left(\frac{2\tilde{\tau }}{\sigma_{w}^{2}}\right),$$

hence, the threshold $\tilde{\tau }$ of the FC can be expressed by

(109) $$\tilde{\tau }=\frac{\sigma_{w}^{2}}{2}F_{2acKN}^{-1}\left(1-\pi_{\mathrm{FA}}\right).$$

Under the hypothesis $H_{1}$, the variable $\tilde{T}$ in (10) is again a chi-squared random variable with 2MS=2acKN degrees of freedom; therefore, the probability of detection of the FC can be expressed as

(110) $$\pi_{D}=\mathrm{Pr}\left\{\tilde{T}\geq \tilde{\tau }|H_{1}\right\}=1-F_{2acKN}\left(\frac{2\tilde{\tau }}{\sigma_{s}^{2}+\sigma_{w}^{2}}\right),$$

hence, the ROC is expressed by

(111) $$\pi_{D}=1-F_{2acKN}\left(\frac{F_{2acKN}^{-1}\left(1-\pi_{\mathrm{FA}}\right)}{1+\gamma }\right),$$

where $\gamma =\sigma_{s}^{2}/\sigma_{w}^{2}$ is the sensing SNR of each ED.

Also, for the performance of the FC with SR, we can use the same approximations used in Section 4.2, i.e., GA, PGA, and PA. Below, we summarize the PGA approach, which includes the GA as a special case when r=1. The analysis could be extended to the PA case with minor modifications, and therefore, for the sake of brevity, we omit the details for the PA case.

The PGA is obtained as

(112) $$F_{2acKN}\left(z\right)\approx 1-Q\left(\left[\sqrt[{r}]{\frac{z}{2acKN}}-\frac{2acKNr^{2}-r+1}{2acKNr^{2}}\right]r\sqrt{acKN}\right),$$

while the FC threshold can be computed as

(113) $$\tilde{\tau }\approx acKN\sigma_{w}^{2}{\left[\frac{Q^{-1}\left(\pi_{\mathrm{FA}}\right)}{r\sqrt{acKN}}+\frac{2acKNr^{2}-r+1}{2acKNr^{2}}\right]}^{r}.$$

Equation (112) permits the expression of the ROC as

(114) $$\pi_{D}\approx Q\left(\frac{Q^{-1}\left(\pi_{\mathrm{FA}}\right)-\left(\sqrt[{r}]{1+\gamma }-1\right)\left(r\sqrt{acKN}-\frac{r-1}{2r\sqrt{acKN}}\right)}{\sqrt[{r}]{1+\gamma }}\right).$$

In this SR case, the product between the total number of sensors *K* and the total number of samples *N* of each ED is bounded by

(115) $$KN\geq \frac{1}{ac}\left({\tilde{b}}_{r}+\frac{r-1}{2r^{2}}+\sqrt{{\tilde{b}}_{r}^{2}+\frac{r-1}{r^{2}}{\tilde{b}}_{r}}\right),$$

where

(116) $${\tilde{b}}_{r}=\frac{1}{2}{\left[\frac{Q^{-1}\left(\pi_{\mathrm{FA}}\right)-\sqrt[{r}]{1+\gamma }Q^{-1}\left(\pi_{D}\right)}{r(\sqrt[{r}]{1+\gamma }-1)}\right]}^{2}.$$

Equation (114) also gives the required SNR γ as

(117) $$\gamma \geq {\left[\frac{Q^{-1}\left(\pi_{\mathrm{FA}}\right)+r\sqrt{acKN}-\frac{r-1}{2r\sqrt{acKN}}}{Q^{-1}\left(\pi_{D}\right)+r\sqrt{acKN}-\frac{r-1}{2r\sqrt{acKN}}}\right]}^{r}-1.$$

In all Equations (112)–(117), the value of *r* can be selected as desired.

## 5. Numerical Results: Validation and Discussion

We use numerical results to validate the performance analysis of Section 4. Specifically, we want to compare the accuracy of the different approximations used in our proposed performance analysis.

**Figure 1:**
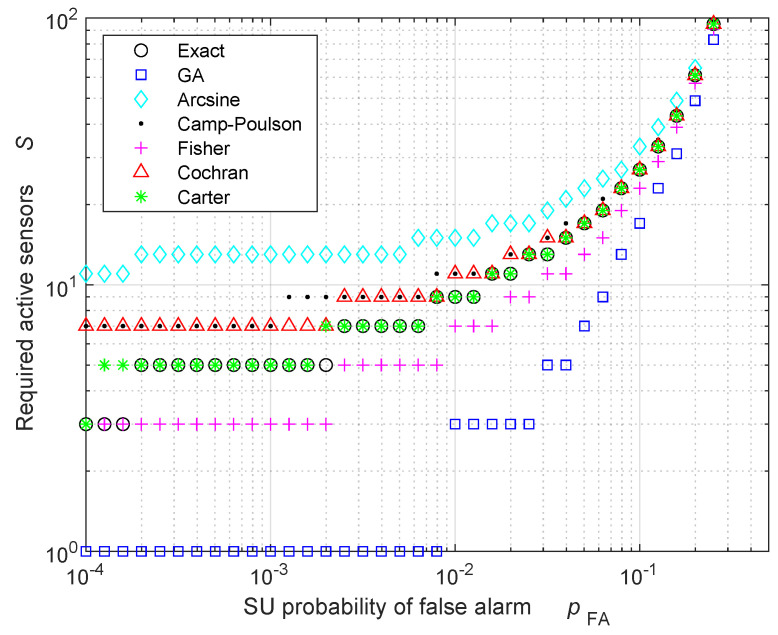
Required number of active sensors to obtain a probability of FA equal to $\pi_{\mathrm{FA}}=10^{-7}$ at the FC in the HR case. All the results are rounded towards the next odd integer.

We start with the FC performance in the HR case. For the *and-rule* and *or-rule* cases, the performance analysis is exact, so there is no approximation to validate. Hence, we focus on the *MV-rule*, where approximations are present. Figure 1 shows the minimum number of required active sensors $S_{\min,\mathrm{FA}}$ as a function of the SU probability of FA $p_{\mathrm{FA}}$ to obtain a probability of FA at the FC equal to $\pi_{\mathrm{FA}}=10^{-7}$. Since the *MV-rule* requires an odd integer number of sensors, $S_{\min,\mathrm{FA}}$ has been rounded accordingly, using the ceiling function. Figure 1 reveals that the Carter approximation (65) provides the best estimate of the exact number of required active sensors. Also, the approximations (53) and (59), derived from Fisher and Cochran studies, respectively, produce good results in agreement with the exact results. On the contrary, the GA (30) underestimates the minimum number of required active sensors, while the arcsine approximation (36) overestimates the number of active sensors. Therefore, the approximations based on the incomplete beta distribution (Fisher, Cochran, and Carter) are more accurate than the approximations based on the binomial distribution (GA, arcsine, and Camp–Poulson). We verified that this behavior is a general result that is also valid for other values of $\pi_{\mathrm{FA}}$, and also valid for $S_{\min,D}$, whose results (not shown in this paper for the sake of brevity) have the same trends as those of $S_{\min,\mathrm{FA}}$ shown in this paper.

**Figure 2:**
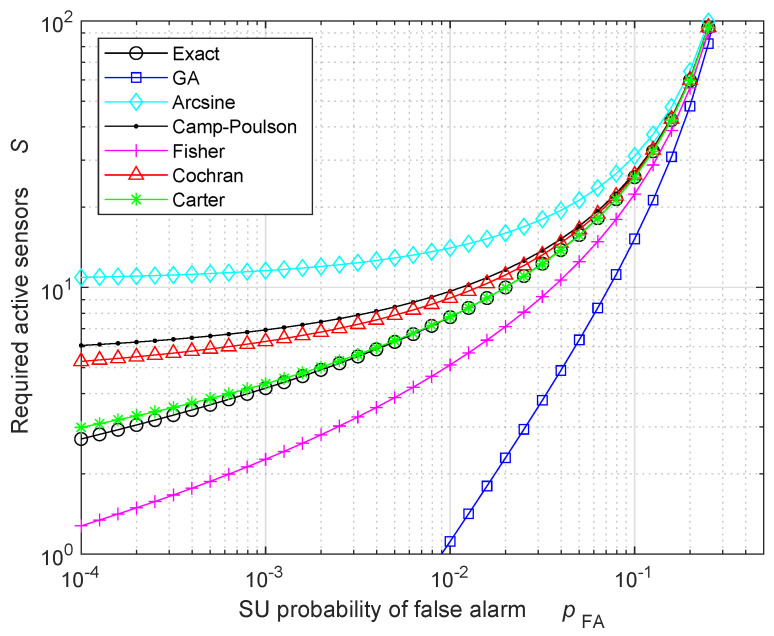
Required number of active sensors to obtain a probability of FA equal to $\pi_{\mathrm{FA}}=10^{-7}$ at the FC, in the HR case. All the results are non-integer.

To better appreciate the accuracy of the different approximations, Figure 2 displays the same approximated performance of Figure 1, but without applying the rounding operation to the next odd integer, leading to a non-integer number of required active sensors $S_{\min,\mathrm{FA}}$. In this case, since the binomial distribution provides integer results, the exact number of required active sensors has been obtained by replacing the binomial distribution with the corresponding incomplete beta distribution, which provides non-integer results. Figure 2 confirms that the Carter approximation provides the best estimate of the required number of active sensors, and that the accuracy of the Carter approximation is greater than those of the other approximations (including Fisher and Cochran ones). Note that the GA provides accurate results only when the exact number of required active sensors is large, as predicted by the CLT. Consequently, the use of a GA performance analysis should be avoided when the number of active sensors is low.

**Figure 3:**
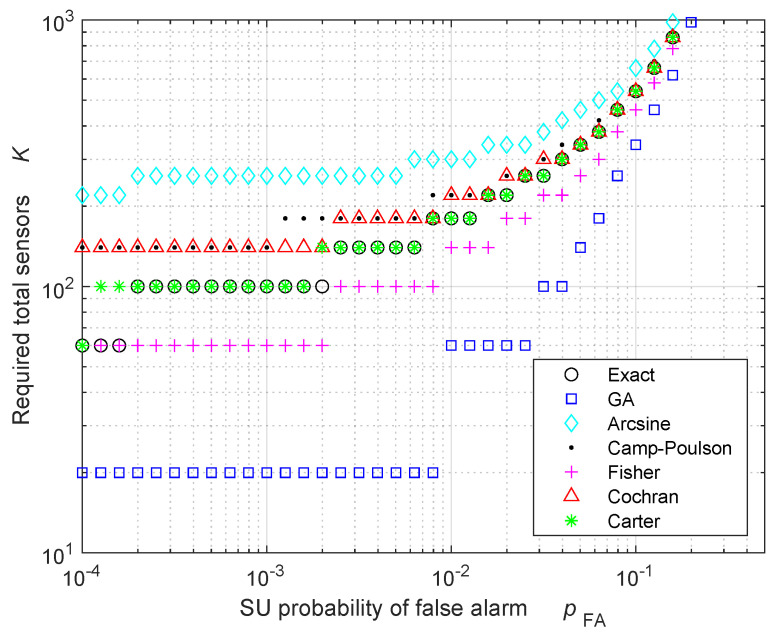
Required number of total sensors to obtain a probability of FA equal to $\pi_{\mathrm{FA}}=10^{-7}$ at the FC, when the activity rate is a=1/20, in the HR case. All the results are rounded such that the number of active sensors is an odd integer.

Figure 3 and Table 1 exhibit the minimum number of total sensors $K_{\min,\mathrm{FA}}$ required as a function of the SU probability of FA $p_{\mathrm{FA}}$, when the activity rate is a=1/20 and the probability of FA at the FC is equal to $\pi_{\mathrm{FA}}=10^{-7}$. The results for the active number of sensors are rounded towards the next odd integer; therefore, all the results in Figure 3 and Table 1 are multiples of 1/a=20. The results in Figure 3 are similar to those in Figure 1, but now the number of required sensors is larger, because most of the sensors are in sleeping mode: when a=1/20, only 5% of the total sensors are active.

**Figure 4:**
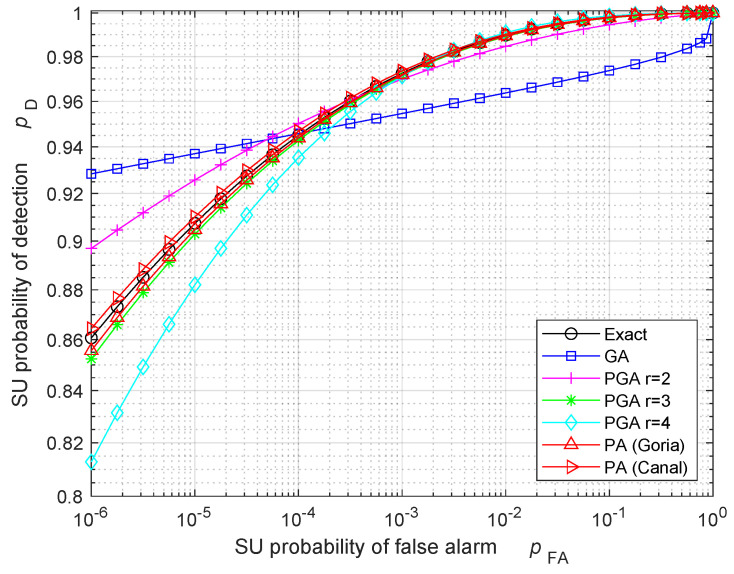
ROC of the SU ED when the number of total samples is N=60, the compression ratio is c=1/10, and the sensing SNR is γ=8 dB.

We now consider the SU performance in the HR case. Specifically, we want to assess the accuracy of GA, PGA, and PA approaches when the SU ED self-estimates its probability of detection and the number of total samples *N*. Figure 4 illustrates the ROC approximations obtained with the different approaches, when the total number of samples is N=60, the compression ratio is c=1/10, and the sensing SNR is γ=8 dB. Figure 4 points out that both the PA approaches give an accurate estimation of the SU probability of detection $p_{D}$. The PGA approach yields a good approximation in the cubic case (r=3), while the other powers (r=2 and r=4) provide less accurate results. The GA is the worst approximation, since the number of compressed samples is too low (M=cN=6) for invoking the CLT. Similar results would be obtained with different values of the number of samples and of the SNR.

**Figure 5:**
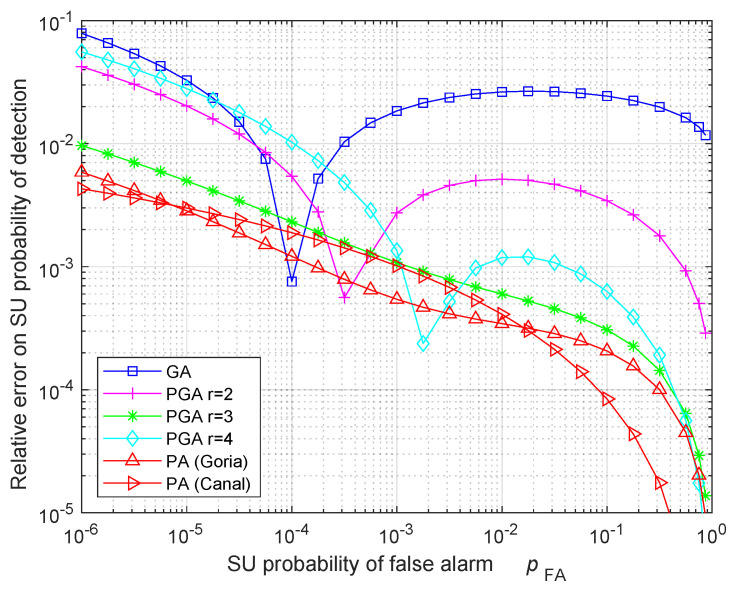
RE on the ROC of the SU ED when the number of total samples is N=60, the compression ratio is c=1/10, and the sensing SNR is γ=8 dB.

Figure 5 highlights the results of Figure 4 by showing the relative error (RE) $\epsilon_{\mathrm{rel}}$ on the ROC, defined by

(118) $$\epsilon_{\mathrm{rel}}=\frac{|p_{D}^{\left(\mathrm{approx}\right)}-p_{D}^{\left(\mathrm{exact}\right)}|}{p_{D}^{\left(\mathrm{exact}\right)}},$$

where $p_{D}^{\left(\mathrm{exact}\right)}$ is the exact probability of detection, and $p_{D}^{\left(\mathrm{approx}\right)}$ is its approximation. Figure 5 gives an idea about the trend of the PA approaches, whose RE reduces for the increasing probability of FA. The same behavior happens for the PGA with r=3, which has a RE below $10^{-2}$ like the PA approaches. On the contrary, the GA and the PGA with r=2 and r=4 have a non-monotonic RE, which can exceed $10^{-2}$ in this scenario. Note that, for some specific values of $p_{\mathrm{FA}}$, the RE shown in Figure 5 can be quite low also for GA and PGA with r=2 and r=4: this behavior only happens for the selected values of $p_{\mathrm{FA}}$, identified by the intersection between the approximated ROC and the exact ROC in Figure 4. Therefore, occasionally, GA and PGA with r=2 and r=4 can produce accurate results, but this accuracy is not generally valid for all the $p_{\mathrm{FA}}$ values.

**Figure 6:**
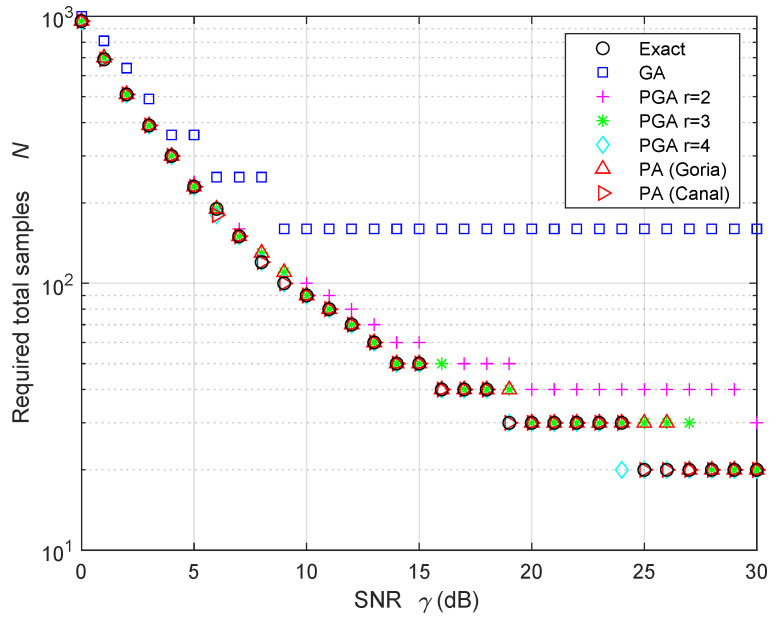
Required number of total samples to obtain a probability of FA equal to $p_{\mathrm{FA}}=10^{-4}$ and a probability of detection equal to $p_{D}=1-10^{-3}$ at the SU, when the compression ratio is c=1/10, in the HR case.

Figure 6 and Table 2 present the results concerning the approximated number of total samples *N* self-estimated by the SU ED when the probability of FA is $p_{\mathrm{FA}}=10^{-4}$, the probability of detection is $p_{D}=1-10^{-3}$, and the compression ratio is c=1/10. The results for the compressed number of samples are rounded towards the next integer; therefore, all the results in Figure 6 and Table 2 are multiples of 1/c=10. In this case, the PA approaches and the PGA approaches with r=3 and r=4 yield accurate estimates of the number of samples *N*. It is worth noting that, in these PA cases, the required number of samples are obtained using the iterative algorithm described in Section 4.2.3, while the PGA results are derived using (84). In addition, note that, at a large SNR γ, the GA largely overestimates the required number of samples, thereby increasing the sensing time more than necessary. This would consequently reduce the capacity of transmission of the SU due to the unnecessary additional time spent for sensing.

**Figure 7:**
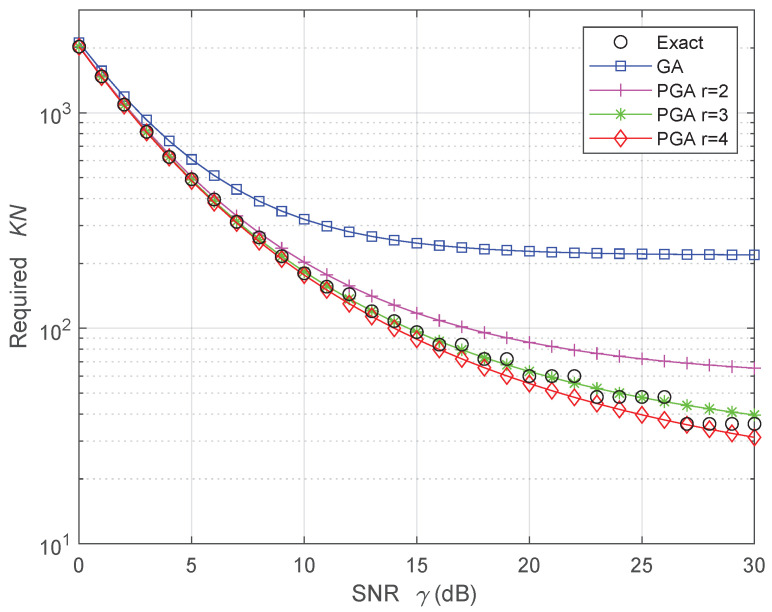
Required product between the number of total sensors *K* and the number of total samples *N* to obtain a probability of FA equal to $\pi_{\mathrm{FA}}=10^{-6}$ and a probability of detection equal to $\pi_{D}=1-10^{-5}$ at the FC, when the activity rate is a=1/4 and the compression ratio is c=1/3, in the SR case. The exact result is rounded such that the number of active sensors times the number of compressed samples is integer, while the approximated values are non-integer.

We now conclude this section by focusing on the SR case. In this case, only the FC performance is of interest, because the SUs do not make any hard decision. Figure 7 underlines the required product KN as a function of the sensing SNR γ, where *K* is the total number of sensors, and *N* is the total number of samples of each SU. An activity rate a=1/4 and a compression ratio c=1/3 are assumed. In this case, the exact result is rounded using the ceiling function, such that the number *S* of active sensors times the number of compressed samples *M* is integer, while the approximated values are non-integer. Figure 7 emphasizes that both PGA approaches with r=3 and r=4 provide accurate results, with minor differences due to the rounding operation. To simplify the readability of Figure 7, the PA results are not shown: in this SR case, an iterative algorithm would produce PA results very close to the PGA results with r=3, similarly to the HR case of Figure 6. Again, the GA approach largely overestimates the product KN, leading to an incorrect estimation with either too many required sensors, or too many required samples per sensor, or both. For instance, when γ=15 dB, the product KN estimated by the GA is more than doubled with respect to the exact value, as shown in Figure 7.

## 6. Conclusions

This paper has proposed useful analytical approximations that characterize the performance of centralized CSS. Specifically, this paper has derived accurate low-complexity closed-form expressions that calculate the required number of sensors and the required number of samples as a function of the probability of detection and the probability of FA of the FC and of the sensors. In contrast with most of the existing literature, this paper does not leverage on the CLT, therefore the proposed analysis is accurate even when the number of active sensors is low and when the sample size is reduced. Therefore, each sensor and the FC can self-compute its own performance with reduced complexity. Future work may extend the proposed approach to sensors equipped with multiple antennas that sense multiple frequency bands.

## Figures and Tables

**Table 1:** Required number of total sensors in Figure 3.

	$p_{\mathrm{FA}}=10^{-4}$	$p_{\mathrm{FA}}=10^{-3}$	$p_{\mathrm{FA}}=10^{-2}$	$p_{\mathrm{FA}}=10^{-1}$
Exact	60	100	180	540
GA	20	20	60	340
Arcsine	220	260	300	660
Camp–Poulson	140	140	220	540
Fisher	60	60	140	460
Cochran	140	140	220	540
Carter	60	100	180	540

**Table 2:** Required number of total samples in Figure 6.

	SNR = 0 dB	SNR = 10 dB	SNR = 20 dB	SNR = 30 dB
Exact	960	90	30	20
GA	1000	160	160	160
PGA, r=2	960	100	40	30
PGA, r=3	960	90	30	20
PGA, r=4	960	90	30	20
PA (Goria)	960	90	30	20
PA (Canal)	960	90	30	20

## Data Availability

The data that support the findings of this study are available from the corresponding author, L.R., upon reasonable request.

## Abbreviations

The following abbreviations are used in this manuscript:

CLT	Central limit theorem
CR	Cognitive radio
CSS	Cooperative spectrum sensing
ED	Energy detector
EGC	Equal-gain combining
FA	False alarm
FC	Fusion center
GA	Gaussian approximation
HR	Hard reporting
MD	Missed detection
MV	Majority voting
PA	Polynomial approximation
pdf	Probability density function
PGA	Power-of-Gaussian approximation
PU	Primary user
RE	Relative error
ROC	Receiver operating characteristic
SNR	Signal-to-noise ratio
SR	Soft reporting
SU	Secondary user
